# Feasibility of a Conversational Agent to Bring Home the STEADI Fall Prevention Algorithm

**DOI:** 10.1093/geroni/igaf122.2632

**Published:** 2025-12-31

**Authors:** Chantal Kerssens, Brenna Phelps, Brian Jones, Madeleine Hackney, Ted Johnson, Joseph (Joe) Nocera, Cathleen Carroll-Sauer, Ritom Gupta

**Affiliations:** Friendi.fi Corporation, Millbrae, California, United States; Georgia Institute of Technology, Atlanta, Georgia, United States; Georgia Institute of Technology, Atlanta, Georgia, United States; Emory School of Medicine, Atlanta, Georgia, United States; Emory School of Medicine, Atlanta, Georgia, United States; Emory University, Atlanta, Georgia, United States; Emory University, Atlanta, Georgia, United States; Friendi.fi Corporation, Millbrae, California, United States

## Abstract

Accidental falls threaten the health and well-being of older adults, especially those with mild cognitive impairment (MCI) due to cognition’s important role in preserving functional gait and postural stability. The STEADI (Stopping Elderly Accidents, Deaths & Injuries) Initiative by the CDC reduces fall risk and rates but traditionally requires extensive, ongoing implementation resources for impact. This study tested the feasibility of using an already-scaled conversational technology platform with tablet-based agent (pet companion avatar) in 21 cognitively-normal (CN) older adults at low fall risk (CN–, n = 6), CN older adults at fall risk (CN+, n = 8), and older adults with MCI (MCI+, n = 7). Fall risk and cognitive status were confirmed with standardized measures. Participants were majority female (76%), 75 ± 10 years old (range 54-95), and racially diverse (White, 62%; Black/African American, 24%; Asian 9%; multi-racial, 5%). All evaluated avatar-led assessments of gait, balance, and leg strength plus an exercise safety check. Users appreciated the avatar’s friendliness, natural conversational tone, nonthreatening design, patience, and engaging nature. Instructions were seen as clear, concise, and easy to follow, corroborating a 9.4 ± 0.8 clarity rating out of 10. System Usability (SU) was good overall (76 ± 13) with areas of improvement noted across groups. Design recommendations included faster response times, clarifying task transitions and starts, and customizing avatar settings such as voice and volume to the individual. The agent and platform are a promising tool to implement STEADI and prevent falls. Future studies will incorporate the Otago Exercise Program.

